# Physical activity and diet for Type 2 Diabetes reduction among older people living with HIV in Harare

**DOI:** 10.4102/hsag.v29i0.2445

**Published:** 2024-02-14

**Authors:** Nongiwe L. Mhlanga, Thinavhuyo R. Netangaheni

**Affiliations:** 1 Department of Health Sciences, College of Human Sciences, University of South Africa, Pretoria, South Africa

**Keywords:** diet, older people living with HIV, physical activity, reduction, Type 2 Diabetes

## Abstract

**Background:**

People living with HIV (PLWH) are ageing, and face increased risk for developing Type 2 Diabetes. Physical activity and diet are effective in reducing Type 2 Diabetes risk. However, there is variation in how older PLWH engage in physical activity and healthy eating.

**Aim:**

To describe older PLWHs’ engagement in physical activity and diet for Type 2 Diabetes risk reduction in Harare

**Setting:**

The study was conducted in five polyclinics in Harare urban district from low socio-economic areas.

**Methods:**

A qualitative approach and an exploratory descriptive design was used. Twenty-three participants were selected purposively based on the researchers’ judgement of age among PLWH. Data were collected using a semi-structured interview schedule and analysed using Braun and Clark’s six steps of thematic content analysis. Ethical approval was obtained, and each participant provided informed consent.

**Results:**

The mean age of participants was 62 years. Participants performed varied physical activities through; economic activities, walking, exercise, and domestic chores. Diet included indigenous, unrefined grains, vegetables and fruit, influenced by rural background. Diet consisted of larger proportions of carbohydrates with lesser varying portions of protein and vegetables and fruit. Healthy eating among participants was facilitated by the proximity of markets and family.

**Conclusion:**

Health education to reduce Type 2 Diabetes risk should focus on fostering current physical activities to meet prescribed standards and increasing fruit and vegetable consumption.

**Contribution:**

The physical activity and diet self-care practices among older PLWH are inadequate in reducing Type 2 Diabetes risk, emphasising the need for appropriate health education.

## Introduction

The use of antiretroviral therapy (ART) for treatment of human immunodeficiency virus (HIV) has improved the life expectancy of people living with HIV (PLWH) (Farahat et al. 2020). With increased life expectancy, older PLWH are at risk of developing chronic illnesses associated with ageing such as Type 2 Diabetes (Farahat et al. 2020). Among PLWH, older age is defined as age 50 years or more, which applies to this study (Thet et al. 2022). Type 2 Diabetes is a chronic metabolic disorder characterised by hyperglycaemia and insulin resistance in the absence of treatment (Goyal, Jialal & Castano 2021). Risks of Type 2 Diabetes include obesity, a first degree relative with Type 2 Diabetes, a history of gestational diabetes, sedentary lifestyle, and Hispanic, Afro-Caribbean and South Asian ethnicity (World Health Organization [WHO] 2020a). Adding to predisposition to Type 2 Diabetes, older PLWH face increased risks because of adverse effects of ART and HIV infection (Noubissi, Katte & Sobngwi 2018). Considering these higher risks, older PLWH ought to practice physical activity and diet self-care to reduce the likelihood of developing Type 2 Diabetes.

The WHO Package of Essential Noncommunicable (PEN) Disease Interventions (2020b) outlines two main self-care strategies to mitigate health risks of Type 2 Diabetes: performing physical activities and adhering to a healthy diet. Substantiating the benefits of physical activity and a healthy diet as self-care to reduce the risk of Type 2 Diabetes, Duncan et al. (2020) conducted a 6-month intervention with older PLWH in the United Kingdom (UK) and found that physical activity and adhering to a healthy diet resulted in weight reduction by 7% and a lowered Homeostatic Model Assessment for Insulin Resistance (HOMA -IR) score of older PLWH. Similarly, Bavaro et al. (2021) assert that self-care practices to reduce weight through diet control and physical activity achieve glycaemic control.

Regarding the implementation of physical activity among older PLWH, the WHO published the first Guidelines for Physical Activity and Sedentary Behaviour in 2020 that included physical activity guidelines for older people living with chronic illness. For older people with chronic illness, WHO (2020b) recommends the regular performance of various physical activities including muscle-strengthening exercises for 2–3 days a week. In addition, moderate-intensity aerobic physical activity is recommended for 150 min–300 min or 75–150 min of vigorous-intensity aerobic physical activity or an equal combination weekly (WHO 2020b).

In light of the increased risks faced by older PLWH, benefits of physical activity and diet as well as recommendations for physical activity by the WHO (2020b), a study in the United States of America (USA) found that older PLWH live highly sedentary lives (Johs et al. 2019). Willig et al. (2021) quantify the finding by Johs et al. (2019) by revealing that in the USA at least 68% of older PLWH do not meet physical activity requirements. Although older PLWH do not meet the physical activity requirements, Voigt et al. (2023) in a qualitative study in an urban setting in the USA found that older PLWH do engage in walking as a self-care practice to maintain physical fitness. Walking in urban areas is more common among older men living with HIV in comparison to women living with HIV, and is hindered by traffic hazards (Voigt et al. 2023). Other physical activity self-care practices are described in a study in Canada conducted by Vader et al. (2017) who found that among older PLWH, physical activities included activities they implemented for daily living.

In Africa, a study in Uganda also found that older PLWH had low levels of physical activity with only 20% of older PLWH meeting physical activity requirements (Wright et al. 2021). Wright et al. (2021) in a description of the types of physical activities engaged by older PLWH found that women engaged in domestic chores such as fetching water, which were of low intensity as part of their physical activity regimes. Moreover, Wright et al. (2021) found that older PLWH engage in a variety of physical activities, which included activities for economic purposes such as farming. Concurring with Wright et al. (2021), in a systematic review of literature from studies in sub-Saharan Africa, Vancampfort, Stubbs and Mugisha (2018) also found that older PLWH have low levels of physical activity. Vancampfort et al. (2018) however differ from Wright et al. (2021) on the issue of economic activities such as farming, by concluding that a lack of economic productivity among older PLWH resulted in lower levels of physical activity. In Mwanza, Tanzania, Kitilya et al. (2023) similar to Wright et al. (2021) found that older PLWH engaged in physical activities through their daily economic activities such as farming and vending of vegetables. In addition to these daily economic activities, Kitilya et al. (2023) concluded that walking was an acceptable form of physical activity among older PLWH. Reiterating the acceptability of walking as a means of physical activity among older PLWH, a South African study conducted in a semi-rural area also found that older PLWH engaged in physical activities through economic activities such as farming, as well as domestic chores (Chetty, Cobbing & Chetty 2022). Furthermore, older PLWH in South Africa engaged in aerobic exercise, particularly in religious groups (Chetty et al. 2022).

Concerning adherence to a healthy diet, Bavaro et al. (2021) and Duncan et al. (2020) found that the Mediterranean Diet was effective in weight reduction to reduce Type 2 Diabetes. The Queensland Government (2021) outlines that the Mediterranean diet is based on the intake of 50% vegetables and fruit, 25% whole grains, 25% fish, poultry or legumes, water as the main beverage; consumption of dairy; use of extra virgin olive, restricted intake of red meat and sugary foods, and eating with others. To reduce Type 2 Diabetes risk, the Mediterranean diet should be modified to suit cultural and individual preferences (Duncan et al. 2020). Despite the benefits of the Mediterranean diet in reducing Type 2 Diabetes risk, a study conducted in Poland found that a quarter of older PLWH consumed whole grains as part of their diet, with most respondents preferring refined forms of carbohydrates such as white rice, pasta and white bread (Duda et al. 2020). Furthermore, only 22.6% of older PLWH consumed fresh fruit and vegetables several times a day (Duda et al. 2020).

In Uganda, Wright et al. (2021) in an observational mixed method study that assessed diet, physical activity and exercise among PLWH in a high-income urban area, concluded that diets of PLWH were homogenous. In a description of the homogeneity of PLWH diets, Wright et al. (2021) found that diets were mostly ‘*matoke*’ a form of banana and sweet potato. The preference for *matoke* according to Wright et al. (2021) was based on the consumption of what was available. Reiterating the issue of lack of diversity in diets of older PLWH Masa, Chowa and Nyirenda (2018) found similar findings in a study conducted in a different context – in rural Zambia. Masa et al. (2018) concluded that the diets of older PLWH were mostly carbohydrates and this was based on socio-economic factors and the staple food available. In South Africa, Oduro and Kissah-Korsah (2021) describe a more diverse diet among older PLWH and conclude that 38.9% of older PLWH had at least three servings of fruit and vegetables with most considering their diet as good.

According to Didisen, Binay and Yardimci (2017), healthcare workers should be able to understand the self-care practised by people. This understanding of people’s self-care practices enables healthcare workers to make decisions regarding current self-care, potential inadequacies, and provide the appropriate interventions such as health education (Didisen et al. 2017). In view of the need to understand self-care physical activity and diet practices among older PLWH, this study was underpinned by Orem’s self-care theory. Smith and Parker (2015) observe that the self-care theory is one of the three minor theories embedded in Orem’s Self-care Deficit Nursing Theory. The theory of self-care is also critical as it enables healthcare workers to design interventions that improve self-care needs of people (Didisen et al. 2017). Three concepts interact to define Orem’s theory of self-care; these are self-care, self-care agency, and self-care requisites (Lambermon et al. 2020:1). Orem (1991) cited in Waligora, Bahouth and Han (2019) refers to self-care as learned behaviours that are outcome focussed performed voluntarily to maintain well-being, life, and health. Smith and Parker (2015) cite Orem (2001) who defines self-care agency as an acquired ability to meet one’s own continuous needs for care that regulate life, health, human development, and well-being. The self-care agency is affected by socio-cultural factors, age, environment, and health. Self-care agency is described in terms of ‘*power*’, ‘*capability*’ and ‘*ability*’. The self-care requisites include the universal, developmental, and health deviation self-care demands, and form the framework for therapeutic self-care demands (Smith & Parker 2015).

Self-care is simply an action for self (Waligora et al. 2019). Orem (2001) cited in Smith and Parker (2015) classifies physical activity and maintaining adequate nutrition as universal self-care requisites.

However, the ageing of PLWH and consequent increased likelihood of developing Type 2 Diabetes among older PLWH described by Farahat et al. (2020) warrants exploration to determine adequacies in reducing Type 2 Diabetes risk. To enable this determination of adequacies of self-care physical activity and healthy diet, yardsticks have been set by the WHO guidelines for physical activity and sedentary life (2020b) as well as studies that have shown the effectiveness of culturally modified Mediterranean diet to reduce Type 2 Diabetes risk by Duncan et al. (2020) and Bavaro et al. (2021). Despite the set standards, for physical activity and diet, a study in Ethiopia found that 61% of older PLWH do not adhere to WHO (2020b) standards of physical activity because of a lack of participation in community engagement activities (Zenu et al. 2023). In light of the inadequate physical activity and dietary patterns by older PLWH reported in Ethiopia by Zenu et al. (2023) and Uganda by Wright et al. (2021) respectively, this study seeks to provide a description of current practices in Harare which enables focused health education where there are inadequacies. Furthermore, studies that have described self-care practices in older PLWH have been conducted in other contexts, creating a paucity of literature from Harare urban district. In this regard, the study seeks to close the population gap of physical activity and diet self-care practices among older PLWH in Harare urban district.

## Aim

The aim of the study was to describe physical activity and diet for Type 2 Diabetes risk reduction among older PLWH in Harare.

## Research methods and design

### Study design

The study used a qualitative approach and an exploratory descriptive study design. Gray and Grove (2021) explain that an exploratory descriptive study design is appropriate for studies that seek to describe a particular situation and cannot be classified as phenomenological, historical, grounded theory or ethnographic. In this case, the situation to be described are the self-care practices of physical activity and diet among older PLWH in Harare urban district.

### Setting

The study was conducted in Harare urban district, one of four districts in Harare province. Participants were recruited from five clinics that provide services to four residential areas in Harare urban district. The catchment areas for the five clinics are densely populated and are classified as low-socio-economic areas (Ncube et al. 2019). Main economic activities in the district include informal trading, urban agriculture and formal employment in manufacturing, agro-processing, construction, health, financial services, and public administration (Chirisa & Mabeza 2019).

### Study population and sampling strategy

The study population in the study were all older PLWH in Harare urban district. The accessible population were the older PLWH accessing ART services in five clinics in Harare urban district.

Inclusion criteria were older PLWH aged more than 50 years willing to take part in the study and accessing ART in the five clinics in Harare urban district.

Exclusion criteria were older PLWH not willing to take part in study, those already diagnosed with Type 2 Diabetes, older PLWH with diminished mental capacity to discern risks and benefits of participating in the study, and older PLWH from institutions such as aged care facilities and prisons who might not have full autonomy to participate in the study.

Purposive sampling was performed to select the sample. The researcher consciously selected the older participants and confirmed their ages with clinic records and self-report. The sample size was determined by data saturation whereby additional recruitment of individual study participants did not yield new data; saturation was reached at 23 participants. Four older PLWH declined to participate citing time constraints on a scheduled ART refill visit. No participants dropped out during data collection.

### Data collection

Data were collected through interviews using a semi-structured interview guide. The interviews were conducted in Shona, interview duration ranged from 19 min-43 min and were conducted from 05 December 2022 till 20 December 2022 then from 16 January 2023 till 13 February 2023. Interviews were audio recorded and conducted at each of the five clinics in a quiet room.

### Data analysis

Data analysis was aimed at deriving an understanding of physical activities and diet of older PLWH for reduction of Type 2 Diabetes risk. Braun and Clark’s six steps of thematic content analysis cited in Campbell et al. (2021) was used to analyse the data. In the first step, collected data were translated to English and transcribed into text, thereafter the text was read to familiarise with the findings. The second step was to generate codes that categorise the data, two main categories emerged. The first category was self-care practices for physical activity where emerging categories included ‘work’ and ‘walking’. The second broad category was self-care practices for diet where emerging categories of data were ‘indigenous’, ‘restricted sugar’ and ‘mainly carbohydrates’. The third step was to organise the initial codes to themes and the initial themes included ‘domestic work and work for economic activities facilitated physical activity’ and ‘diet includes indigenous unrefined grains’. The fourth step was to review the generated themes and review the data to ensure there was ample data to support the generated themes, in this review additional data on ‘indigenous fruit and vegetables’ was added to the theme ‘diet is composed of indigenous unrefined grains’ to support the theme. The fifth step was to define the themes and aligning them to the research question ‘what physical activity and diet self-care practices older PLWH engage in to reduce Type 2 Diabetes risk in Harare urban district’. The sixth step was to describe the findings in a narrative format.

### Trustworthiness

To ensure trustworthiness of the study results, the researchers abided by the framework described by Guba and Lincoln (1994) cited in Polit and Beck (2020). Four indicators of trustworthiness were conformed to, and these were dependability, credibility, transferability, and confirmability. To ensure credibility, the researchers confirmed the study’s findings with the participants. Dependability and transferability were ensured by detailing the steps used to conduct the study and through thick descriptions of the population, setting, data collection, and analysis method. To maintain confirmability, the researchers remained objective in collecting and analysing data and reflected on the researchers’ experience in working with PLWH with comorbid Type 2 Diabetes.

### Ethical considerations

Ethical approval to conduct the study was obtained from the College of Human Sciences Research Ethics Review Committee (CREC reference number 14056739_CREC_CHS_2022). Permission was also granted from the City of Harare, Health Department. To abide by the principle of respect for human dignity, older PLWH rights to full disclosure and self-determination were respected. In upholding the right to full disclosure, older PLWH were individually informed of the purpose of the study, contact details of the researchers, why they had been selected to participate, the benefits of health education provided during the study, the risks of discomfort because of prolonged sitting they may experience, and the right to withdraw and ask questions any time during the interviews. Written consent was obtained from all participants after they had been informed about the study. In upholding the right to self-determination participants were informed that participation was voluntary. Moreover, participants were informed that they would not be prejudiced from any services rendered at the clinics should they decline to participate in the study. The study also considered the principle of justice whereby participants’ names were not used, and all data collected were kept confidentially in a password locked computer only accessible to researchers. To uphold the right to fair treatment, the study did not include older PLWH who were from institutions such as prisons and aged care facilities who may have diminished autonomy to participate in the study. The third principle considered during the study was the principle of beneficence, which included the right to minimise harm and maximise benefits. To minimise harm, participants were informed that they could discontinue with the interviews anytime should they be uncomfortable and they were assured that data collected would be for research purposes only.

## Results

### Participants’ characteristics

A total of 23 older PLWH participated in the study, all participants were black Africans. Table 1 shows sample demographic variables.

**TABLE 1:** Sample demographic variables.

Participant number	Age (years)	Gender	Marital status	Occupation	Duration of HIV infection and ART	Presence of chronic illness
1	53	Male	Widowed	Self-employed	4	Hypertension
2	62	Male	Married	Non-specified	12	-
3	53	Male	Married	Information Technology officer	16	-
4	56	Male	Divorced	Piece jobs in farming	9	-
5	52	Female	Cohabiting	Buying and selling	6	-
6	66	Female	Married	Farmer	6	-
7	64	Male	Married	Non-specified	12	-
8	78	Female	Widowed	Retired	23	Hypertension and arthritis
9	54	Female	Married	Self-employed	12	Hypertension
10	72	Male	Married	Retired	17	-
11	63	Male	Married	Burial society coordinator	14	-
12	51	Male	Married	Self-employed electrician	9	-
13	53	Female	Widowed	Self-employed	6	-
14	67	Female	Widowed	Clothing vendor	11	-
15	64	Female	Widowed	Retired	19	-
16	69	Male	Married	Retired	11	Hypertension
17	72	Male	Married	Retired and farming	21	-
18	69	Female	Married	Retired	15	Hypertension
19	70	Male	Divorced	Retired	12	-
20	56	Female	Divorced	Office orderly	4	Hypertension
21	67	Female	Widowed	Farmer	7	-
22	54	Female	Married	Homemaker	6	-
23	61	Male	Widowed	Driver	8	-

HIV, human immunodeficiency virus; ART, antiretroviral therapy.

### Emerging themes

From the data analysed, six overarching themes emerged. Three themes were aligned to the performance of physical activities, and three were aligned to diet as a self-care measure to reduce Type 2 Diabetes risk. The themes emerged in response to the questions: What is your physical activity routines on a weekly or daily basis? What dietary self-care measures do you implement to maintain a healthy diet in an effort to reduce Type 2 Diabetes? What proportions of carbohydrates, oils, sugary foods, fruit, vegetables and protein do you consume daily? and What elements in your environment, socio-cultural background have enabled the implementation of physical activity and diet self-care practices?

#### Theme 1: Economic activities facilitate physical activity

The participants described how their economic activities were physically involving and this enabled self-care for physical activity. Participant 4 explained that he is a manual labourer who mostly does farming work for 8–9h daily. Participant 11 shared his experience as a burial society coordinator whose work involved cycling long distances between residential suburbs to coordinate burial society activities as part of his economic productivity. Participant 21 described how her farming work for farming beans for sale kept her physically active. Participant 5 shared that they bought and sold household wares and this involved a lot of travelling, which was physically involving. Given below are the excerpts from participants’ experiences:

‘I work manually for 8–9 hours a day, I do piece jobs which are manual, so we have to meet work targets, to get the money, we get into the field in the morning and leave at 5 pm.’ (Participant 4, 56-year-old male, divorced)

‘I exercise through my farming work, we plough the fields, dig, and plant and till the land and we harvest and carry the harvest physically to our home we then have time to rest, that is how I exercise through my work, I grow beans and sell them.’ (Participant 21, 67-year-old female, widowed)

‘I work very hard, that’s why I am fit and can run faster than you, I carry the goods I sell and travel to different places to sell.’ (Participant 5, 52-year-old female, cohabiting)

The theme of economic activities facilitating physical activity self-care was described by participants involved in different occupations.

**Subtheme 1.2:** Self-care physical activities include domestic chores: The issue of domestic chores as a form of physical activity was also shared by the participants. Participant 8 described how she had no one to assist her and she cooked, cleaned, swept and did some gardening. It is worth noting that Participant 8 was disabled and walked with the aid of crutches. Participant 22 shared that she maintained her garden. Participant 22 acknowledged that although she did the domestic chores, the work she did was not regular. Participant 15 shared her experience of looking after her grandchildren, and this work was physically exerting as it involved cleaning, preparing children for school and cooking. The quotes from the participants’ shared experiences are as follows:

‘I work in my house, I have no helper, I clean my dishes, I still can work with my upper body, I am only disabled waist downwards, I tend to my garden, I sweep outside I use two walking canes as I sweep my house, I do laundry.’ (Participant 8, 78-year-old female, widowed)

‘I maintain my garden and the yard around my house that is the physical activity I do, but it is not regular.’ Participant 22, 54-year-old female, married)

All participants who described the issue of domestic work as a form of physical activity to reduce Type 2 diabetes development were females. The participants described the different activities they performed, and these included cooking, cleaning, and gardening.

**Subtheme 1.2:** Physical activity includes routine walking. The participants shared their experiences of walking as part of the self-care routine to maintain physical fitness. Participant 3 described how he walked at least 10 km every weekend to do their soccer betting, as their occupation during the week involved a lot of sitting and the weekend was an opportunity to engage in physical activity. Participant 20 also described how she walked part of the distance to their place of work as a means of maintaining physical activity, in sharing the experience Participant 20 revealed that she walked a ‘long distance’. It is noteworthy that walking habits of Participants 3 and 20 were influenced by the economic activities they were engaged in. Although their walking was not influenced by economic activities, Participant 2 also described that they walked around their house twice a day in the morning and evening as part of their physical activity routine. The quotes from participants are as follows:

‘Looking at my age when it comes to jogging, I don’t jog, I try as much as I can walk, I leave the car, and walk 5km going and 5 km coming back, I walk mostly weekends to do my soccer betting, it is that distance so I feel I should go on foot (Participant 3, 53-year-old male, married)

‘I go to work that’s how I exercise; I walk part of my journey to work, from town to the suburb my workplace is located, I walk, then I come back using a car, it is quite a distance to that street so that is my exercise.’ (Participant 20, 56-year-old female, divorced)

Male and female participants described that walking was a routine physical activity they engaged in as a means of ensuring physical fitness. Two of these participants observed that their economic activities motivated the walking.

#### Theme 2: Physical activity self-care practices include following an exercise routine

Participants described how they followed structured exercise routines, weekly or daily. Participant 1 explained that they ran for 30 min–40 min twice a week. Participant 2 shared they walked twice a day and performed push-ups daily as an exercise routine. Participant 12 also described that they played social soccer once every week and as part of the routine they performed various exercises. Excerpts from shared experiences from Participants 1 and 12 to support theme 2 are as follows:

‘When I finish my breakfast in the morning, I walk around my house, and a few hours later I exercise in the house by doing push-ups and before I sleep, I exercise by walking around the house again.’ (Participant 2, 62-year-old male, married)

‘With all the sitting I will be doing during the week, I have to do some exercising, so I play social soccer, it is not serious with my friends from another township, we have been doing it for some time and it is once every week. We get there we warm up with different exercises before we play.’ (Participant 12, 51-year-old male, married)

‘I feel the need to exercise the body, so I run 30 to 40 minutes twice every week.’ (Participant 1, 53-year-old male, widowed)

The participants who described following an exercise routine were all males. These participants also described different frequencies with which they performed their exercises with Participant 1 and 12 observing that they exercised weekly and Participant 2 sharing that they exercised daily.

#### Theme 3: Self-care includes performance of varied physical activities

The third theme to emerge was that self-care includes the performance of varied physical activities. Participant 3 in sharing their experience described how he walked at least 5 km every weekend to do their soccer betting and would also engage in farming activities for part of the year during the summer farming season (October–February). Similarly, Participant 11 stated that his physical activities included cycling as well as working in the garden. Participant 20 who had described how she walked as part of her journey to her workplace also stated that her occupation as an office orderly included a variety of other physically involving activities. The participants shared their experiences as follows:

‘Normally I exercise through the work I do with my hands; I work in the garden. I ride my bicycle to and from one township to another, so naturally that is my exercise that is my means of transport which helps me stay strong, I do it daily as I can be called to any burial society in another township, to another I will be on my bicycle, I do not use a taxi.’ (Participant 11, 63-year-old male, married)

From the participants shared experiences, older PLWH perform various physical activities, which involve different groups of muscles for example Participant 11 used legs for cycling to work and their hand for gardening.

Theme 4–7 below describe the self-care practices pertaining to diet in the reduction of Type 2 Diabetes risk among older PLWH.

#### Theme 4: Diet includes indigenous whole grains, vegetables and fruit

The participants shared their experiences of eating indigenous whole grains, indigenous vegetables, and fruit as part of their diet. Participant 13 outlined that she ate the Southern African porridge (*sadza*) (Collins English Dictionary 2023) made from finger millet or sorghum as well as pumpkin leaves (*muboora*) and amaranth leaves (*bonongwe*): Participant 23 shared that their diet included indigenous vegetables such as pumpkin leaves. Participant 6 like Participant 13 also explained that her diet included varieties of the Southern African porridge made from finger millet and sorghum. Participant 17 described their preference of indigenous fruits such as Uapaca kirkiana (*mazhanje*), as well as okra and pumpkin leaves:

‘I eat traditional vegetables a lot like pumpkin leaves, I supplement them with peanut butter, I also take some tea every day and usually eat bananas, mangoes and apples.’ (Participant 23, 61-year-old male, widowed)

‘I eat Southern African porridge [*sadza*] made from ground sorghum [*rapoko*], or finger millet [*mhunga*] I also eat a lot indigenous vegetables like pumpkin leaves [*muboora*] and amaranth leaves [*bonongwe*], which are healthy for me.’ (Participant 13, 53-year-old female, widowed)

Some participants who described the consumption of indigenous whole grains also shared that their preference for eating the indigenous foods was influenced by their rural backgrounds. This influence of rural background on a diet of indigenous whole grains, fruit and vegetables emerged as a subtheme to theme 4 and was supported by excerpts from Participants 6, who observed that they grew up in rural Gutu in Masvingo province and their diet included Southern African porridge made from sorghum or finger millet and they also ate vegetables like amaranth leaves, black jack (*mutsine*), pumpkin leaves and okra (*derere*), which they grew up eating. Similarly, Participant 17 shared that they grew up in a rural area and explained that their diet which included indigenous whole grains and vegetables was influenced by the previous rurality. The excerpts are as follows:

‘I don’t normally select the milled grain I use, I grew up I rural areas we ate all types of Southern African Porridge [*sadza*], so I eat all types of Southern African Porridge. I eat Southern African Porridge made from sorghum, finger millet, I grew up in Gutu in Masvingo Province where, the finger millet Southern African Porridge was the most common one which is what I eat most of the time now, in terms of vegetables I eat amaranth leaves [*bonongwe*], blackjack [*mutsine*] and okra [*derere*] we eat what we grew up eating.’ (Participant 6, 66-year-old female, married)

‘If it is Southern African Porridge [*sadza*] I make sure the maize meal is unrefined, I also eat natural indigenous fruit like Uapaca kirkiana [*mazhanje*] because I grew up in the rural areas, I eat bananas and apples less than five times a year, I also eat a lot of mangoes. When it comes to vegetables, during this planting season I eat indigenous vegetables like pumpkin leaves [*muboora*] and amaranth leaves [*bonongwe*], I even drink the water from cooking them, I also eat a lot of okra [*derere*] I spend the whole week eating okra as part of my meal.’ (Participant 17, 72-year-old male, married)

#### Theme 5: Diet is mostly carbohydrates, with varied lesser portions of vegetables, fruit and proteins and restricted use of oils, salt and sugar

The participants further shared that their diets were mostly carbohydrates in the form of Southern African porridge, rice, and pasta. The participants estimated that this portion of carbohydrates was 50%. This was described by Participant 12 who noticed that their daily meals include a larger proportion of starch in the form of Southern African porridge, rice, and pasta from breakfast, lunch till dinner, with 20% vegetable intake throughout the meals. Participants further estimated that their diets were 20% fruit and vegetables, and 30% protein or vice versa. The consumption of higher proportions of carbohydrates was also explained by Participant 17. In addition, Participant 8 also described how her diet was mostly Southern African porridge with about 10% protein with little sugar in their tea and restricted oils. Participant 1 also shared that their diet was mostly Southern African porridge (50%) and about 10% fruit and restricted use of salt as they had been educated about high salt intake. It is noteworthy that Participant 1 self-reported that they were hypertensive. Quotes to support theme 4 are shown as follows:

‘I eat a normal diet, Southern African Porridge with meat and fruit from time to time, about 10% of the diet is fruit and 50% is Southern African porridge and vegetables, I restrict my salt intake because we have been taught about controlling our blood pressure, and I use a little sugar because it is my preference.’ (Participant 1, 53-year-old male, widowed)

‘I would give 50% to Southern African porridge, protein is 20%, vegetables are 30%; but sometimes vegetables are 20% and protein is 30%. I do not use a lot of oil in my food and with sugar it is just a few granules, just so people can say I have used the sugar.’ (Participant 17, 72-year-old male, married)

From the participants’ descriptions, it was concluded that diet was mostly carbohydrates in the form of Southern African porridge, which is characteristic of staple food in Zimbabwe. The participants shared they consumed lesser but varying portions of fruit and vegetables, from 10% and up to 30%, and protein sources were included in lesser proportions to carbohydrates.

#### Theme 6: Family support and easy access to fruit and vegetables foster self-care for healthy eating

The last theme to emerge was that family support and easy access to fruit and vegetable markets foster self-care for healthy eating. Participant 1 stated that they had easy access to fruit and vegetables like tomatoes, apples, bananas, and vegetables from the nearby market. Participant 3 also shared that he easily had access to fruit and vegetables from the market and their spouse assisted them with the selection of healthy food. Participant 10 shared that fruit vendors move from door to door selling fruit which made it easy to access fruit and vegetables. Participant 8 also stated that despite her disability they could easily reach the supermarket and had access to healthy food from their garden which was also a source of physical activity. Furthermore, Participant 8 observed that her grown-up children supported them financially enabling them to buy healthy food. Participant 6 similarly shared that she had financial support from their grown-up children, which fostered the self-care for healthy eating. The quotes to support theme 6 are shown as follows:

‘Every weekend I go with my wife to Mbare market [*common market*] and she picks out the potatoes, tomatoes, onions and fruit.’ (Participant 3, 53-year-old male, married)

‘Where I stay a lot of vendors sell vegetables in push carts, some knock on the door to sell to us, so this access makes it easy for us.’ (Participant 10, 72-year-old male, married)

‘With my work in farming, I can get the food, however, my children also support me financially which helps me supplement the food I get.’ (Participant 6, 66-year-old female, married)

## Discussion

The study sought to describe physical activity and diet self-care practices for the reduction of Type 2 Diabetes risk among older PLWH. The study found that economic activities among older PLWH facilitated physical activities. These findings are confirmatory of findings from the mixed method observational study conducted in an urban setting in Uganda by Wright et al. (2021) who found that economic activities among older PLWH facilitated physical activities. The study also affirms the findings of Kitilya et al. (2023) who similarly used a qualitative approach in Mwanza Tanzania and found that older PLWH do engage in physical activities through economic activities such as farming and vending. It is noteworthy that, the issue of vending as a facilitator of physical activity described by Kitilya et al. (2023) was elaborated by Participant 5 in this study who described how buying and selling household wares enabled physical fitness through travelling. Kitilya et al. (2023) notably make the conclusion in a setting where sources of livelihood included fishing, farming, livestock production, and mining.However, the finding that economic activities facilitated physical activity in this study contrasts with findings from a systematic review of studies from sub-Saharan Africa by Vancampfort et al. (2018) who found that older PLWH did not meet physical activity requirements as they were not economically active. It is notable that the findings from this study found that despite most participants (30%) stating that they were retired, some participants such as Participant 21 explained that although she was retired, she was physically active by engaging in economic activities such as farming. The issue of farming activities in an urban context also confirms the sprawling urban agriculture characteristic of the Harare urban district described by Chirisa and Mabeza (2019).

Beyond economic activities facilitating physical activity among older PLWH, the study also found that domestic activities enabled physical activity. This finding was shared by only female participants. This finding is confirmatory of findings made in different contexts: Vader et al. (2017) in Canada; Wright et al. (2021) in Uganda; Chetty et al. (2022) in a semi-rural location in South Africa. Despite this confirmation that domestic activities enable physical activity, the study conducted by Wright et al. (2021) measured the intensity of domestic chores among participants and concluded that domestic chores were of low intensity to meet physical activity requirements. The conclusion by Wright et al. (2021) that domestic activities are of low intensity to meet physical activity requirements for older PLWH implies a need for further investigation in Harare urban district on the intensity of domestic activities reported by the participants in this study.

Duncan et al. (2020) illustrated the effectiveness of walking for the reduction of Type 2 Diabetes risk among older PLWH. This study in alignment found that older PLWH walked as a means of physical activity. The acceptability of walking among older PLWH is also reiterated in the study by Voigt et al. (2023) in an urban area in the USA. Voigt et al. (2023) also observed that men were more likely to walk as a means of physical activity in comparison to women. Voigt et al. (2023) further revealed that walking among older PLWH was impeded by traffic hazards. In contrast to Voigt et al. (2023), this study found that both men and women walked as a means of physical activity. Furthermore, the desirability of walking as a means of physical activity was illustrated by findings from this study as older PLWH described how they engaged in various physical activities, which included walking. As such, health education should foster adequate levels of walking to reduce Type 2 Diabetes risk. Regarding the performance of various physical activities, the study further confirms the findings by Wright et al. (2021) who also found that older PLWH performed varied physical activities.

The study also described the self-care practices of older PLWH in relation to diet as a means for reducing Type 2 Diabetes risk. From the data analysed, the study found that the diet included consumption of indigenous grains, vegetables, and fruit. This consumption of indigenous food was influenced by rural background. These findings although unique to Southern African contexts, are not unexpected as other studies also report that people consume diets that are culturally acceptable. For example, Wright et al. (2021) describe the consumption of ‘*matoke*’ a form of banana and sweet potato in Uganda, which is a diet indigenous to that context. Duncan et al. (2020) in their study found that diet intervention included individualisation of diet, and this was performed through dietary advice that suits ethnicity. This implies the need to provide health education that suits the consumption of indigenous food especially for older PLWH whose choice to consume indigenous food was influenced by their rural background.

Commendably the consumption of unrefined grain included in the diet of older PLWH in Harare urban district aligns with recommendations of a Mediterranean diet described by the Queensland Government (2021), which recommends that 25% of carbohydrates consumed should be wholegrains. This finding differs from the Polish results by Duda et al. (2021) who found that most respondents consumed ‘*over milled*’ forms of carbohydrates such as white bread and white rice. Such variation could partly be attributed to the difference in geographical settings of the studies and the consequent influence of ethnicity on food choices.

The participants described that their diet consisted mostly of carbohydrates with varying proportions of fruit, vegetables, and protein. Similar findings are described from the study conducted in Zambia by Masa et al. (2018) who found that diets in older PLWH consisted of mostly carbohydrates, and this was attributed to available staple foods and affordability. Comparatively, the study in South Africa by Oduro and Kissah-Korsah (2021) revealed that 38.9% of older PLWH consumed at least three servings of fruit and vegetables daily while in Poland Duda et al. (2021) found that 22.9% of older PLWH consumed fruit and vegetables several times daily. The lower proportions of fruit and vegetables reported by the participants in this study are undesirable to reduce Type 2 Diabetes risk based on findings by Duncan et al. (2020) and the description provided by the Queensland Government (2021). This consumption of lower proportions of fruit and vegetables could potentially negate the benefits yielded from the consumption of unrefined indigenous grains described by participants in this study. As such there is a need for healthcare workers to provide education that fosters increased consumption of fruit and vegetables. It is noteworthy that adherence to health education fostering increased consumption of fruit and vegetables could also be supported by the proximity of fruit and vegetables that are available in markets and gardens described by the participants.

The study was underpinned by Orem’s self-care model; from the findings, the universal self-care requisites to maintain health and well-being included performing various physical activities, walking, consumption of indigenous unrefined grains, fruit, and vegetables. The performance of diet self-care requisites of eating unrefined grains related to the self-care agency’s abilities, which were in turn positively influenced by the sociocultural factor of a previous rural background. On the other hand, the economic activities and domestic chores were a means of providing the self-care agency with the capabilities for physical activity and enabled the performance of universal self-care requisites of walking and engaging in various physical activities. The study also found that the therapeutic self-care demand for health education to increase the consumption of fruit and vegetables among older PLWH to reduce Type 2 Diabetes risk.

## Conclusion

The study concludes that for older PLWH in Harare urban district physical activity self-care practices for the reduction of Type 2 Diabetes risk are through their engagement in economic activities, domestic chores, walking, and these physical activities are varied. Regarding dietary self-care practices, older PLWH consume unrefined indigenous grains, fruit and vegetables. The diet is mostly carbohydrates with varying but lesser proportions of fruit vegetables and protein. In view of these results, there is a need to provide health education that fosters increased consumption of fruit and vegetables and ensures older PLWH continue the engagement in physical activities. While the researchers made an effort to ensure accurate results, the study was limited by the inability to measure the intensity of physical activity reported by participants. As such it is recommended that additional research addresses the intensity of physical activities among older PLWH to enable accurate judgement whether the activity patterns are sufficient to meet required levels to reduce Type 2 Diabetes risk. Moreover, further research can be performed to accurately quantify the proportions of each nutrient intake taken, as the study used estimates provided by the older PLWH.
